# An Empirical Study of the Effects of Incidental Vocabulary Learning Through Listening to Songs

**DOI:** 10.3389/fpsyg.2022.891146

**Published:** 2022-05-18

**Authors:** Kaihua Nie, Jing Fu, Hina Rehman, Ghulam Hussain Khan Zaigham

**Affiliations:** ^1^Foreign Language School, Hubei Engineering University, Xiaogan, China; ^2^Faculty of Management Science, National University of Modern Languages (NUML), Islamabad, Pakistan; ^3^Department of Management, Comsats University Islamabad, Islamabad, Pakistan

**Keywords:** incidental vocabulary learning, English songs, vocabulary knowledge, frequency of exposure, prior vocabulary knowledge

## Abstract

Most studies have shown that reading is an important source of incidental vocabulary learning, and repeated reading may have a positive effect on learning gains. However, the study of incidental vocabulary learning through listening is still limited, and the immediate and long-term effects on different vocabulary knowledge dimensions are unclear. Furthermore, no empirical studies have been conducted to investigate the association between learning gains and preexisting vocabulary knowledge in listening. This article examines the effects of listening to English songs on unintentional vocabulary learning and vocabulary retention through three different vocabulary knowledge dimensions: word recognition, meaning association, and grammar identification. A total of 114 Chinese college students participated in the study, and they were given vocabulary evaluations at different times based on three separate components of vocabulary knowledge. The effects of repeated listening (one, three, and five times) and learners’ prior vocabulary knowledge were also investigated. According to the findings, listening to songs can improve vocabulary knowledge, particularly in the area of word recognition, which can be retained 4 weeks later. Furthermore, the effect of listening three times (with exposure frequencies ranging from three to nine) was superior than listening one or five times, which provides teachers and learners with guidance for teaching or learning vocabulary more effectively. Finally, for low, intermediate, and high-level learners, there was an immediate and positive effect on the dimensions of word recognition and meaning connection after listening, and this knowledge is likely to be preserved 4 weeks later.

## Introduction

Learning vocabulary has long been regarded as an indispensable component of mastering a second language. According to Schmitt (2008), a large vocabulary is required to function in English, and the lexical numbers for both reading and oral discourse set to be learned should be high. However, due to the short amount of time available for instruction, students are unable to acquire large numbers of vocabulary items in the classroom (Cunningham, 2005). So, social media can be considered an alternative language learning technique for learners, particularly for those who live in monolingual societies (Kerekes, 2014; Bano et al., 2019; Kakar and Khan, 2020; Raza et al., 2020; Khan, 2021b). As Schmitt (2000) study pointed, vocabulary can be acquired either intentionally or incidentally; students should take full advantage of incidental lexicon learning, too. Given the importance of vocabulary in language learning, there have been numerous studies on accidental vocabulary learning in second language acquisition (e.g., Dupuy and Krashen, 1993; Horst et al., 1998; Paribakht and Wesche, 1999). According to these studies, the vast majority of researchers (e.g., Brown et al., 2008; Hatami, 2017) have looked at the extent to which vocabulary information can be addressed by reading, whereas few researchers have looked at the acquisition of incidental vocabulary through listening (Vidal, 2011; Van Zeeland and Schmitt, 2013; Hatami, 2017). For example, Vidal (2011) discovered that listening to academic lectures can result in incidental vocabulary learning. Furthermore, Van Zeeland and Schmitt (2013) demonstrated a positive effect by listening to a variety of spoken input sources, including talk shows, television interviews, and informal lectures. Regardless of the approaches to listening described above, listening to songs also plays a significant role in our lives, and songs can supply large amounts of language input (Schwarz, 2013). Nonetheless, apart from Medina’s (1993), Maneshi’s (2017), and Pavia et al.’s (2019) studies, no empirical studies have been conducted on listening to L2 songs as a method for vocabulary learning.

The current study focused on incidental vocabulary acquisition through listening to two pop songs and attempted to see if three areas of vocabulary knowledge (word recognition, meaning connection, and grammar) were retained 4 weeks after exposure, which was a drawback of prior listening studies. Additionally, because people like to listen to the same music repeatedly (Lems, 2001; Tegge, 2018), the impact of listening to the same song (one, three, or five times) on learning gains was investigated. Besides, for learners with a range of lexical sizes (Khan and Khan, 2019), the association between diverse prior vocabulary knowledge and incidental vocabulary growth was also examined.

## Literature Review

### Vocabulary Knowledge

Despite the need to know many vocabulary items, learners must also understand the “depth” of vocabulary knowledge, which is just as essential as the number of items (Schmitt, 2008). Lexicon knowledge is multifaceted, and there are various sorts of lexical components that can be acquired (Schmitt, 1994; Nation, 2013). For instance, orthography, syntax, grammar, collocation, and meaning recall are all independent components of lexicon knowledge (Webb, 2005, 2007). However, vocabulary learning is an incremental process, and different components of vocabulary knowledge may develop in different ways (Schmitt, 1994). For example, a recent empirical study by Teng (2018) examined the consequences of reading while listening, *via* graded readers acquiring different dimensions of vocabulary knowledge (word recognition, grammar identification, meaning associations, and collocations), and the findings revealed 65, 43, 30, and 20% gains, respectively. While vocabulary knowledge is a crucial part and reliable factor of learners’ proficiency in second language learning, most studies have focused on reading, and little research has addressed this issue with regard to listening (e.g., Bonk, 2000).

#### Incidental Vocabulary Learning

When learners unintentionally expand new aspects of their L2, this is known as incidental learning (Van Zeeland and Schmitt, 2013). The acquisition of new vocabulary information through accidental learning, also known as incidental vocabulary learning, has long aroused the curiosity of researchers (Van Zeeland and Schmitt, 2013). Generally, incidental vocabulary learning is the understanding of by-product activity, which is not specifically planned to teach vocabulary (Hulstijn, 2001). As Webb and Nation (2017) point out, learning vocabulary incidentally may occur when encountering various L2 spoken and written discourse inputs that enable learners’ vocabulary growth. However, the majority of research on accidental vocabulary learning has focused on acquiring new words through reading, such as graded readers, stories, newspapers, textbooks, etc., and there is a consensus that reading is an effective way to expand one’s vocabulary (e.g., Krashen, 1989; Rott, 2007). For example, Webb and Chang (2015a) improved the effectiveness with 44.06% of recognition, and 36.66% of meaning recall after reading ten graded readers. Furthermore, Pellicer-Sánchez and Schmitt (2010) investigated form recognition, grammatical class recall, and meaning association while reading an authentic novel. They found that learners could perceive the meaning and form for 84 and 76% of words, individually, but remembering the meaning and word class for 55 and 63% of words. Moreover, social and mobile media also have been mentioned as a source of learning and performance outcome in past studies (Pitafi et al., 2018; Ali et al., 2019; Khan, 2021a; Khan et al., 2022).

### The Role of Audio Input in Incidental Vocabulary Learning

Listening also helps in the acquisition of accidental vocabulary (Brown et al., 2008). For example, Medina’s (1993) research was an initial empirical study to explore L2 vocabulary development through listening. It was found that children’s songs can help with L2 vocabulary development, but the amount of lexicon learned from listening to songs is not specified, and the dimensions of vocabulary knowledge are not mentioned. Furthermore, Vidal (2011) asserts that listening to academic lectures might result in accidental vocabulary learning. In an immediate posttest, considerable learning improvements of 15.5% were observed by listening, and learning growth of 7.8% remained 4 weeks later. As English as a Foreign Language (EFL) learners do not have a wide range of input environments to choose from, academics are increasingly interested in unintentional vocabulary learning by listening to sources of knowledge that are accessible to learners in circumstances with restricted input, especially in a more entertaining way. For example, Van Zeeland and Schmitt (2013) study looked into several types of spoken input, including audio talk shows, interviews, and informal lectures. Overall, both immediate and 2-week delayed tests revealed vocabulary acquisition growth of 29.2% (7.05 words) and 19% (4.58 words), respectively. Furthermore, Pavia et al.’s (2019) research found that listening to L2 songs contributed to vocabulary learning and repeated listening had a positive effect on vocabulary gains. However, prior vocabulary knowledge was not taken into account in the study, which found a strong link between vocabulary size and vocabulary gains (Vidal, 2003, 2011; Peters et al., 2016). Besides, a recent experimental study looked into incidental vocabulary acquisition through song listening, and the results showed that learners can acquire vocabulary knowledge very immediately after listening, particularly in the dimension of word recognition. However, the delayed posttest results indicated an increase from the immediate posttest across both experimental and control groups, which cannot be used to guide or contribute to future research (Maneshi, 2017). What is more, song lyrics can benefit L2 learners by exposing them to forms, syntax, lexical items, segments, and suprasegmentals (Abbott, 2002). As a result, it is important to investigate the magnitude of incidental vocabulary learning through listening to songs, particularly for language learners in low-input environments.

### The Role of Frequency of Exposure in Incidental Vocabulary Learning

Frequency of exposure is a popular research area that has gained many researchers’ attention, and the results vary across different studies. Most studies illustrate that the more frequently learners are exposed to target words, the more likely they are to be learned (Reynolds and Wible, 2014). Moreover, Maneshi (2017) research also found that repeated listening improved vocabulary knowledge gains, with the effect of listening five times outperforming listening once and three times, especially in the areas of word recognition and meaning association. As Penno et al.’s (2002) study points out, participants could attain more vocabulary items as the number of encounters increased in a listening to a story activity, and learners were able to utilize words more precisely in a story retelling task. However, Webb and Chang (2015a) findings for the association between number of occurrences and vocabulary acquisition differ from those of prior studies (Saragi et al., 1978; Horst et al., 1998; Webb, 2007; Vidal, 2011). Webb and Chang (2015a) surprisingly found that vocabulary gains did not have a strong relationship with the number and distribution of occurrences in reading. The posttest relationship between relative gain and exposure frequency (*r* = −0.03, *p* = 0.78, *n* = 100) was determined to be relatively low and non-significant. The connection between relative gains and exposure was not statistically significant in a delayed posttest (*r* = −0.17, *p* = 0.09, *n* = 100). This is probably because the time between reading and testing was relatively long, ranging from one to 13 weeks. The findings of Webb and Chang’s study appear to support Nation and Wang (1999) assertion that no one number of exposures could guarantee acquisition, and the link between exposure and vocabulary learning is complicated by a variety of additional factors (Saragi et al., 1978). For example, to investigate the association between word understanding and exposure frequency, learners may require different frequencies of exposure to assimilate different types of vocabulary knowledge. Furthermore, in Pavia et al.’s (2019) study, the researchers found that listening to songs repeatedly improved the vocabulary knowledge of word recognition, meaning connections, and collocations, however, the acquisition of vocabulary knowledge in meaning connection and collocation may be less than word recognition. Moreover, Pigada and Schmitt (2006) assert that the meaning recognition of a word requires a wider range of encounters than just spelling.

### The Role of Prior Vocabulary Knowledge in Incidental Vocabulary Learning

Some researchers found a strong relationship between learner’s prior vocabulary knowledge and learning gains in incidental vocabulary learning. For example, Murphy et al.’s (2021) study indicated that, due to prior vocabulary knowledge disparities, the achievement of incidental vocabulary learning varied among learners in the activity of reading. Besides, other researches have also claimed that students with greater vocabulary knowledge benefit more from incidental vocabulary learning while reading (Pulido, 2003, 2007; Webb and Chang, 2015b). However, due to the fact that vocabulary learning increases in listening are substantially smaller than in reading (e.g., Vidal, 2011), academics have paid less attention to the topic of the link between learners’ preexisting vocabulary knowledge and learning achievement through listening. There were little studies looked into the relationship between learner’s prior vocabulary sizes and learning gains. For example, Vidal (2003, 2011) research found a favorable association between vocabulary size and learning growth through academic listening and repeatedly reading. What is more, other researchers found that learners with higher vocabulary sizes being more likely to learn new words than those with smaller vocabulary sizes through watching television (Peters et al., 2016; Peters and Webb, 2018). One the contrary, Rodgers (2013) study found no evidence that learners with bigger vocabulary sizes could obtain more vocabulary from watching television than learners with smaller vocabulary sizes.

### The Present Study

To date, while research has shed light on the quantity and quality of vocabulary learned *via* reading, empirical evidence for how listening aids L2 learners’ vocabulary acquisition through hearing is still lacking. Songs can provide a lot of verbal input (Schwarz, 2013). As a result, the aim of this study was to look into this further and see if there was a link between the amount of L2 learners’ vocabulary and their vocabulary knowledge aspects by listening to songs. Spoken-form recognition, grammar recognition, and meaning recall are the three dimensions of vocabulary knowledge that will be measured. Furthermore, people like to listen to the same songs repeatedly (Tegge, 2018), and the number of encounters with unfamiliar words may affect incidental learning gains (Webb and Chang, 2015a), so the effect of repeated listening to songs (one, three, or five times) on learning gains was also investigated.

#### Research Questions

To what extent does listening to songs increase vocabulary knowledge of spoken-form recognition, grammar recognition, and meaning recall?

To what extent does frequency of exposure affect incidental vocabulary learning through listening to songs?

To what extent does participants’ prior vocabulary knowledge affect incidental vocabulary learning through listening to songs?

## Methodology

### Participants

The participants were 114 Chinese EFL learners who were first-year undergraduate students, and their ages ranged from 18 to 20 years. The participants had similar educational backgrounds, and all of them had studied English for at least 9 years, with a similar level of proficiency generally. In order to analyze the relationship between participants’ previous vocabulary size and incidental lexicon gains, all students completed a Vocabulary Level Test (VLT), and the vocabulary test results indicated that almost all the students were familiar with the first 4,000 words, 25% of the students less than the first 4,000 words (low level), 48% of the students from 4,000 to 5,900 words (intermediate level), and 27% of students more than the first 6,000 words (high level). The students were reassigned into four groups: a control group, E1 (listening to songs once), E3 (listening to songs three times), and E5 (listening to songs five times). Each group contained a similar number of students, with eight high-level students, 14 intermediate-level students, and seven low-level students, respectively.

### Instruments

#### Listening Materials: English Songs

The research’s learning material consisted of two English songs. The first song was Jason Mraz’s “Have it all” (Song A), while the second song was Billy Joel’s “Piano Man” (Song B). When choosing songs and target words, a number of factors were taken into account. First and foremost, it was critical to ensure that the participants had not heard the songs before. This contributed to the study’s validity by ensuring that learning improvements could be assigned to the learning circumstances. Second, the singers of both songs needed to have accurate pronunciation and a consistent beat, so song genres like Rap and R&B were avoided. Third, learners’ existing vocabulary had to account for 95% of vocabulary for optimal acquisition.

The lyrics of the songs are 593 and 283 words long, respectively. There were 876 words in all. Range (Heatley and Nation, 2002) and Nation’s (2017) British National Corpus/Corpus of Contemporary American English (BNC/COCA) word family lists were used to examine the lyrics of the two songs. Range analysis determines the vocabulary level of each song at a certain level based on the lexical frequency of its phrases (Nation and Webb, 2011). According to the analysis, the participants needed to know at least the most frequent 2,000 word families to achieve around 93% lexical coverage of the songs, but their lexical levels were more than 2,000 frequent words, putting them in the best position for unassisted incidental vocabulary learning (see Tables 1, 2).

**TABLE 1:** Lexical frequency profile: “Have it all.”

Word list families	Tokens/%	Types/%
One-thousand (157)	829/89.14	184/72.73
Two-thousand (30)	34/3.66	30/11.86
Three-thousand (2)	4/0.43	2/0.79
Off-lists	63/6.77	37/14.62
Total (189)	930	253

**TABLE 2:** Lexical frequency profile: “Piano man.”

Word list families	Tokens/%	Types/%
One-thousand (111)	268/88.16	132/79.52
Two-thousand (13)	13/4.28	13/7.83
Three-thousand (1)	1/0.33	1/0.60
Off-lists	22/7.24	20/12.05
Total (304)	166	125

#### Test of Prior Vocabulary Knowledge

The participants took an English and Chinese language version of the VLT to assess their past vocabulary knowledge. There are 140 multiple-choice questions in all, with 10 questions from each 1,000-word family level. The 140-item test is effective because it covers a wide range of frequency levels, comprises a large number of items, and the items have been meticulously prepared. To determine learners’ entire receptive vocabulary size, their total score is multiplied by 100. The following website has bilingual versions.^1^

#### Target Words

Twenty-six target words and 13 distractors were included for two songs. The inclusion of familiar terms was intended to encourage participants to complete the entire exam rather than give up when they realized that the majority of the words were unknown. Due to grammar identification being one form of vocabulary knowledge investigated during the tests, the vocabulary types in the choices had to be in various forms, such as nouns, verbs, and adjectives. However, adverbs were not utilized as target words because they are straightforward to identify with others. The 13 target words appeared 1–3 times on average, with word levels ranging from 2,000 to 7,000. Even though there were some vocabularies from lower word levels, none of the target words had been learned by the participants prior to the study (see Table 3).

**TABLE 3:** Frequency of occurrence, and word level of target words.

Song A	Frequency	Word level/1,000	Song B	Frequency	Word level/1,000
Esteem	1	6	Shuffle	1	5
Paved	2	3	Tonic	1	7
Infinite	3	3	Gin	1	6
Slap	1	4	Melody	2	4
Bracelet	1	7	Estate	1	2
Clutter	1	6	Carnival	1	6
Chaos	1	4			

*The 13 distractors were as follows: deficit, condense, beneficial, accelerate, resent, peculiar, prospect, adjust, commence, sensible, radical, asset, and barrier.*

### Vocabulary Knowledge Tests

Following Webb’s (2005, 2007) and Pavia et al.’s (2019) testing design, there were 78 multiple-choice items included in total. Throughout the whole study, there were three tests included to follow and compare the development of the students: a test before listening, a test immediately after listening, and a test 4 weeks later. Each assessment contained three sections that identify different areas of vocabulary knowledge, such as recognition, meaning recall, and grammar. Due to the fact that listening to songs is an aural activity, the measurements were also presented in aural form to suit listening behavior. The participants were instructed to listen to the recordings for questions and alternatives in multiple-choice assessments and then choose their answers from an answer sheet. Each question contained four alternatives: one correct answer, two distractors, and one “I don’t know” choice. When creating distractors, a number of factors were taken into account. First, the words are usually confused with original ones by most students. Second, the parts of speech for each word should be comparable. Third, for recognition distractors, the initial letters of words are generally modified to make them seem like actual English terms. Before starting the study, all the instructions and examples were explained in Chinese (native language) to ensure that all participants fully understood the test.

#### Spoken-Form Recognition

The first component of the test focused on single-word item identification in spoken form. This section included seven and six multiple-choice items for Song A and Song B, respectively. Besides, the remaining 13 multiple-choice items were designed to encourage students to use words they already knew. All the distractors were nonsense words, which were created by altering the initial letters of words to make them sound like real English terms, but easily confused by students since they shared similar parts of speech and syllables to the target vocabulary. For this section, all participants listened to the tape once, with a 2-s stop between choices and a 4-s pause between items. The time break for each choice and item was adapted from Van Zeeland and Schmitt (2013) study. An example question is presented below:

##### Spoken-Form Recognition Testing

###### The Participant Sees on Paper. 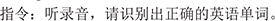


A. B. C. D. I don’t know

The participant hears on the recording:

“No.1 [1 second] A, esteemed [2 seconds] B, gasteemed [2 seconds] C, presteemed [2 seconds].”

#### Form-Meaning Connection

A recall exam was used to assess knowledge of meaning in the second section. It was chosen because it assesses students’ comprehension of listening, and the test looked at the connection between form-meaning and single-word items. This part also contained seven and six multiple-choice items for two songs individually, and the rest were 13 multiple-choice items to motivate the students with words they were previously familiar with. Participants were then asked to tick the relevant Chinese translations of the alternatives on the answer sheet after hearing the target words for the first time. The distractors were responses to other exam items. The participants were given ten seconds for each item, with a four-second interval between them. An example question is presented below:

##### Form-Meaning Connection Testing

###### The Participant Sees on Paper. 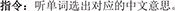


1). A.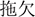 B.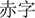 C.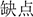 D. I don’t know

At the same time the participant hears on the recording

“Number one [1 sec.] Shuffle.”

#### Grammar Recognition Testing

A multiple-choice test was used in the third section to assess receptive knowledge of grammatical functions. Song A had 7 multiple-choice items, Song B 6 multiple-choice items, and the rest of the 13 multiple-choice items were to motivate students with words they already knew. The structure of the testing followed Webb’s (2005, 2007) research. Students were asked to tick the corresponding part of speech for each item, such as a noun, verb, or adjective, on a piece of paper as well as in a recording. Since adverbs are easily recognized, there was no adverb option supplied as an alternative. Furthermore, three sentences with target words were provided after each item, and students were asked to identify the sentence with correct grammar. The function of a short sentence for each option is to help participants comprehend how a noun, verb, or adjective can be used in context. Each item was allotted six seconds, giving participants adequate time to recognize the grammar of words. The following is an example question:

##### Grammar Recognition Testing

###### The Participant Sees on Paper. 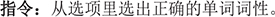


1). Deficit (a) It is a deficit. (b) It deficits. (c) It is very deficit.

The participant hears a recording at the same time.

“No.1 [1 sec.] deficit.”

### Procedure

Participants (except the control group students) listened to the songs and performed assessments in one-on-one sessions. In week 1, all the participants were required to complete a bilingual version of the VLT (English and Chinese) at the outset, and they were assigned to three different levels of students (high, intermediate, and low-level students). As a result, preexisting classes were divided into four groups in total, which were one control group (C) (*n* = 29) and three experimental groups: E1 (*n* = 29), E3 (*n* = 29), and E5 (*n* = 27), each with similar proportions of high, intermediate, and low-level students. Next, all the participants (including control group students) finished a pretest for two songs before listening.

In week 2, the experimental participants were asked to listen to the songs once at the beginning, and they were told that they would be asked some questions related to the songs afterward. The purpose of the listening comprehension test was to ensure that all of them could comprehensively understand the context of the songs and make incidental vocabulary learning happen. Next, after listening to two songs, the experimental group (E1) was required to complete a posttest immediately. Finally, the experimental groups (E3 and E5) differed in the number of times they listened to the music, with the E3 group listening three times and the E5 group listening five times. The control group, on the other hand, had to complete the immediate posttest without listening to the songs.

Four weeks later, all participants took a delayed posttest for both songs.

### Data Analysis

The data were analyzed using SPSS (Version 25). Non-parametric tests were performed to address the study questions as a Kolmogorov–Smirnov test gave a negative result for a normal distribution. To answer these three study questions, all data were scored dichotomously, with 0 representing an incorrect answer and 1 a correct answer. For the first research question, a Mann–Whitney U Test was used to compare results within each group (control and experimental) on three dimensions (recognition, meaning, and grammar) at various times of testing (pretest, immediate posttest, and delayed posttest). To evaluate the relationship between vocabulary knowledge (recognition, meaning, and grammar) and the frequency of exposure (one time, three times, and five times) participants listened to the songs accordingly, and repeated Kruskal–Wallis Tests were performed. Finally, repeated Friedman M Tests were used to investigate the link between vocabulary size and the three dimensions of vocabulary knowledge (recognition, meaning, and grammar) after listening to the songs in order to address the third study question.

## Results

Tables 4–6 show descriptive data for vocabulary learning gains, frequency of exposure, and prior vocabulary knowledge of each aspect during vocabulary assessments. In order to address these three research questions, repeated measurements from Mann–Whitney U Tests, Kruskal–Wallis Tests, and Friedman M Tests were used, respectively, to compare the results for three dimensions of vocabulary knowledge within control and experimental groups at various testing times.

**TABLE 4:** Descriptive statistics for vocabulary knowledge.

Time of testing	*N*	Mean	SD	Minimum	Maximum	25th	50th (median)	75th
Recognition								
1	114	3.80	1.896	0	8	2.00	4.00	5.00
2	114	6.57	3.001	0	13	5.00	6.00	8.25
3	114	6.36	2.618	1	12	4.00	6.50	8.00
Meaning								
1	114	2.69	1.834	0	8	1.00	2.00	4.00
2	114	4.15	2.301	0	10	2.00	4.00	6.00
3	114	4.86	2.534	0	12	3.00	5.00	6.25
Grammar								
1	114	6.52	2.179	0	12	5.00	7.00	8.00
2	114	6.67	2.209	0	13	5.00	7.00	8.00
3	114	7.27	2.088	0	11	6.00	7.00	9.00
Groups	114	0.75	0.437	0	1	0.00	1.00	1.00

*Test time: 1, retest; 2, immediate posttest; 3, delayed posttest.*

**TABLE 5:** Descriptive statistics for frequency of exposure.

						Percentiles		
	*N*	Mean	SD	Minimum	Maximum	25th	50th (median)	75*^th^*
Immediate posttest								
Recognition	114	6.57	3.001	0	13	5.00	6.00	8.25
Meaning	114	4.15	2.301	0	10	2.00	4.00	6.00
Grammar	114	6.67	2.209	0	13	5.00	7.00	8.00
Frequency	114	2.20	1.910	0	5	0.00	1.00	3.00
Delayed posttest								
Recognition	114	6.36	2.618	1	12	4.00	6.50	8.00
Meaning	114	4.86	2.534	0	12	3.00	5.00	6.25
Grammar	114	7.27	2.088	0	11	6.00	7.00	9.00
Frequency	114	2.20	1.910	0	5	0.00	1.00	3.0

**TABLE 6:** Descriptive statistics for prior vocabulary knowledge.

							Percentiles		
	Time of testing	*N*	Mean	SD	Minimum	Maximum	25th	50th (median)	75th
Low-level									
Recognition	1	20	2.35	1.631	0	6	1.25	2.00	3.75
	2	20	4.55	2.585	0	9	2.25	4.50	6.75
	3	20	4.70	1.922	1	8	3.25	4.50	6.50
Meaning	1	20	1.45	1.099	0	5	1.00	1.00	2.00
	2	20	3.15	1.644	0	6	2.00	3.00	4.00
	3	20	3.75	2.268	1	11	2.00	3.00	5.00
Grammar	1	20	6.00	1.717	3	10	5.00	6.00	7.00
	2	20	6.20	1.673	3	9	5.00	6.00	7.00
	3	20	7.05	2.089	2	10	6.00	7.00	8.00
Intermediate-level									
Recognition	1	41	3.98	1.666	0	7	3.00	4.00	5.00
	2	41	7.54	2.847	3	13	5.00	6.00	11.00
	3	41	6.90	2.478	1	11	5.00	7.00	9.00
Meaning	1	41	2.46	1.614	0	6	1.00	2.00	3.50
	2	41	4.59	2.291	0	9	3.00	4.00	6.00
	3	41	4.59	2.398	0	10	3.00	5.00	6.00
Grammar	1	41	6.80	1.990	2	11	5.00	7.00	8.50
	2	41	6.66	2.198	2	10	5.00	7.00	8.00
	3	41	7.39	1.986	3	11	6.00	8.00	9.00
High-level									
Recognition	1	24	4.71	1.829	1	8	4.00	5.00	6.00
	2	24	8.75	2.674	4	13	7.00	8.50	11.00
	3	24	8.04	2.562	3	12	7.00	8.00	10.75
Meaning	1	24	4.21	2.064	1	8	2.25	4.00	5.75
	2	24	5.92	2.358	2	10	4.25	5.00	8.00
	3	24	7.04	2.293	2	12	5.00	7.00	8.75
Grammar	1	24	7.25	2.327	4	12	5.00	8.00	9.00
	2	24	8.25	2.172	4	13	7.00	8.00	9.75
	3	24	7.78	2.092	3	11	7.00	8.00	9.00

*Test time: 1, pretest; 2, immediate posttest; 3, delayed posttest.*

### The Effect of Listening to Songs on Incidental Vocabulary Learning

The first study issue addresses the extent to which word recognition, grammar identification, and meaning associations of vocabulary knowledge are addressed by listening to English songs. For each knowledge category, independent samples of Mann–Whitney U Tests were used to compare scores on a pretest, immediate posttest, and delayed posttest. The findings revealed that there was a significant difference in the immediate posttest (*z* = −3.725, *p* = 0.001) and the delayed posttest (*z* = −2.465, *p* = 0.014) for spoken-form recognition, but no significant difference in the pretest. For the form-meaning connection of the test, there was a significant difference in the immediate posttest (z = −3.783, *p* < 0.001), but no significant difference in the pretest and delayed posttest. For grammar recognition, there was a significant difference in the immediate posttest (*z* = −2.673, *p* = 0.008), but no difference in the pretest and delayed posttest. Overall, the three aspects of vocabulary knowledge showed significant differences after listening to the songs immediately, and the difference in spoken-form recognition was more significant than form-meaning connection and grammar recognition, with data for the percentiles being 15.39, 7.69, and 7.7%, respectively. However, only spoken-form recognition revealed a significant difference in the delayed posttest, whereas form-meaning connection and grammar recognition showed no significant differences (see Table 7).

**TABLE 7:** Mann–Whitney U Test statistics.

	Spoken-form recognition			Form-meaning connection			Grammar recognition		
Participant subgroups	Pretest	Immediate posttest	Delayed posttest	Pretest	Immediate posttest	Delayed posttest	Pretest	Immediate posttest	Delayed posttest
Control (percentiles)	4 (2.5–5)	5 (4–6)	5 (4–6.5)	3 (1.5–4)	3 (2–4)	4 (3–6)	6 (4–8)	6 (5–7)	7 (6–8)
Experimental (percentiles)	4 (2–5)	7 (5–10)	7 (4–8.)	2 (1–4)	4 (3–6)	5 (3–7)	7 (5–8)	7 (5.5–8.5)	8 (6–9)
*Z*	−0.056	−3.725	−2.465	−0.285	−3.783	−1.551	−1.591	−2.673	−1.602
Significant	0.955	0.000195	0.014	0.776	0.000155	0.121	0.112	0.008	0.109
									

### The Effect of Frequency of Exposure on Incidental Vocabulary Learning

The second study question concerns the impact of exposure frequency on incidental vocabulary learning through music listening. According to the immediate posttest hypothesis test, repeated Kruskal–Wallis tests revealed that all three aspects of vocabulary knowledge exhibited essential differences, with *p* < 0.001 in each case. The results of frequency recognition pairwise comparisons revealed a significant difference in frequency from 0 to 3 times only (*p* < 0.001), with percentiles of 5.00 and 8.00, respectively (see percentiles in Table 8). In the meaning pairwise comparisons of frequency, the data showed that there were critical differences from 0 to 3 times (*p* < 0.001), 0 to 5 times (*p* = 0.005), and 1 to 3 times (*p* = 0.002), with the same percentiles from 3.00 to 5.00. In the grammar pairwise comparisons of frequency, the findings showed that there were significant differences from 0 to 3 times (*p* = 0.001), with percentiles from 6.00 to 8.00, and 5 times to 3 times (*p* = 0.014), with percentiles from 6.00 to 8.00. However, according to the post-delayed test hypothesis test, only recognition rejects the null hypothesis (*p* = 0.003), with the frequencies of 0 to 3 times and 5 times to 3 times exhibiting significant differences at *p* = 0.004 and *p* = 0.030, respectively. In the recognition pairwise comparisons of frequency, the data showed that there were significant differences from 0 to 3 times and from 5 to 3 times, with the same percentiles from 5.00 to 8.00 (see percentiles in Table 8).

**TABLE 8:** Percentiles for frequency.

Frequency	Immediate posttest	Delayed posttest
	Percentile (mean)	Percentile (mean)
Recognition		
0 time	5	5
1 time	6	7
3 times	8	8
5 times	6	5
Meaning		
0 time	3	4
1 time	3	5
3 times	5	5
5 times	5	5
Grammar		
0 time	6	7
1 time	7	7
3 times	8	8
5 times	6	8

### The Effect of Prior Vocabulary Knowledge on Incidental Vocabulary Learning

The third research question investigated how participants’ pre-existing vocabulary knowledge influenced incidental vocabulary learning while listening to songs. Friedman M Testsrevealed that between a pretest, immediate posttest, and delayed posttest, there was a statistically significant difference in vocabulary knowledge of spoken-form recognition for low, intermediate, and high level students, with (Chi-square = 11.676; *p* = 0.003), (Chi-square = 41.883; *p* < 0.001), and (Chi-square = 19.976; *p* < 0.001), respectively. Furthermore, for post hoc comparisons, a Bonferroni test was used for spoken-form recognition, and the results showed that all low, intermediate, and high level students showed a statistically significant difference between the pretest and immediate posttest (*p* = 0.022; *p* < 0.001; *p* < 0.001), between the pretest and post delayed test (*p* = 0.017; *p* < 0.001; *p* = 0.003), but no significant difference between the immediate test and post delayed test (*p* = 1.000; *p* = 1.001; *p* = 1.000). For vocabulary knowledge of meaning connections, low, intermediate, and high level students’ hypothesis results also all indicated an overall statistically significant difference between the pretest, immediate posttest, and delayed posttest with (Chi-square = 20.366; *p* < 0.001), (Chi-square = 34.503; *p* < 0.001), and (Chi-square = 20.447; *p* < 0.001), respectively. Also, post-hoc comparisons used a Bonferroni test for meaning connection, and the results showed that all low, intermediate, and high level students indicated a clear difference between a pretest and an immediate posttest (*p* = 0.001; *p* < 0.001; *p* = 0.003), a pretest and a post delayed test (*p* < 0.001; *p* < 0.001; *p* = 0.001), but no significant difference between an immediate test and a post delayed test (*p* = 1.000; *p* = 1.000; *p* = 1.000). However, for vocabulary knowledge of grammar, low, intermediate, and high level hypothesis results showed no statistically significant difference between a pretest, immediate posttest, and delayed posttest with (Chi-square = 2.893; *p* = 0.235), (Chi-square = 1.972; *p* = 0.373), and (Chi-square = 5.297; *p* = 0.071), respectively.

## Discussion

### To What Extent Does Listening to Songs Increase Vocabulary Knowledge of Spoken-Form Recognition, Grammar Recognition, and Meaning Recall?

In terms of the first research question, the findings revealed that listening to songs resulted in substantial gains in three dimensions (recognition, meaning, and grammar) in an immediate posttest, while only spoken-form recognition demonstrated significant acquisition in a delayed posttest. This finding is partly consistent with that of Van Zeeland and Schmitt (2013). Both studies found that participants advanced in three aspects of knowledge, recognition > grammar > meaning, immediately after listening to songs. A first possible reason for learners’ preference for spoken-form recognition over meaning connection is that spoken-form recognition is the first and most straightforward dimension to master after listening. According to Van Zeeland and Schmitt (2013), the acquisition of meaning recall requires a greater number of exposures than spoken-form recognition. Second, knowledge of meaning connections is a very complex element, since most vocabulary words have more than two meanings, and these meanings vary in different contexts, which makes it difficult for learners to identify and recall them after listening.

However, given the result of the delayed posttest, the current finding is not consistent with previous studies of incidental vocabulary retention of three vocabulary knowledge aspects (e.g., Van Zeeland and Schmitt, 2013). The present study found that both meaning recall and grammar recognition showed no significant difference in a delayed posttest, while spoken-form recognition did reveal a significant difference 4 weeks later. Nevertheless, Van Zeeland and Schmitt (2013) research found that meaning recall showed no obvious difference between an immediate posttest and a delayed posttest, although there was attrition of both grammar and recognition over the course of 2 weeks. These variations in findings are most probably related to time interval differences, because the current post-delayed test duration is substantially longer than in Van Zeeland and Schmitt (2013) investigation, so meaning recall and grammar were almost lost 4 weeks later. Generally, these findings suggest that learners can learn the spoken forms and meaning forms, and perform grammar recognition of single-word items incidentally while listening to songs. However, except for spoken-form recognition, which remained at 15.39% 4 weeks later, vocabulary knowledge of meaning and grammar were not easily retained.

### To What Extent Does the Frequency of Exposure Affect Incidental Learning Through Listening to Songs?

In answer to the second study question, the findings revealed that three dimensions of vocabulary knowledge demonstrate positive effects after listening to songs repeatedly. According to the findings, there was a strong correlation between the number of exposures and learning increases for all three dimensions after listening to songs three times, with the frequency of exposure to target words ranging from three to nine times.

The findings of this study are similar to those of earlier research. Pavia et al. (2019) study also found a strong association between the number of exposures and learning gains for spoken-form identification after listening to songs three times, where the frequency of exposure to target words ranged from three to 18 times. However, the current study unexpectedly found that listening five times revealed no lexical improvement for experimental groups. There are probably two reasons: first, many factors may affect learners’ vocabulary acquisition, such as learners’ motivation, concentration, the complexity of words, etc., and frequency is only one of these. So, repeated listening might improve vocabulary knowledge, but this does not imply that the larger the more times a song is heard, the greater the total amount of vocabulary information obtained. As a result, listening to songs five times did not have the positive effect as most people expected. Second, the time taken to listen to two songs five times was quite long (over 30 min), and students already knew they would listen to the songs five times at the outset. According to the researchers’ observation, most participants did not really concentrate on the songs while listening, and they may have paid more attention to the music than the lyrics. As a consequence, when the number of times students listened to songs was raised to five, their vocabulary knowledge did not continue to grow, and their recognition and grammatical learning improvements were nearly identical to those of the control group (zero times). Furthermore, the results of the post-delayed test revealed that only vocabulary knowledge of recognition showed retentive memory for students who listened to the songs three times, and knowledge of meaning connections and grammar recognition showed no difference between a pretest and a post delayed posttest for all students after 4 weeks.

In general, listening to English songs three times increases L2 vocabulary knowledge in the areas of word recognition, meaning connection, and grammatical recognition, with word recognition information likely to be preserved after 4 weeks. However, when the number of times that learners listen to songs rises, students become distracted and find it harder to concentrate on vocabulary items while listening, resulting in little vocabulary knowledge being attained.

### To What Extent Does Participants’ Prior Vocabulary Knowledge Affect Incidental Vocabulary Learning Through Listening to Songs?

In response to the third study question, the data imply that low-proficiency, intermediate-proficiency, and high-proficiency learners all showed significant achievement in word recognition and meaning recall after listening to songs, which lasted for 4 weeks, but there was no progress in grammar recognition. There are several possible explanations for the positive findings for word recognition and meaning recall for all levels of students. To begin with, spoken-form recognition is easier to acquire than form-meaning connections and grammar (Van Zeeland and Schmitt, 2013). Besides, listening to music is a joyful activity that can motivate students (Richards, 1969; Tegge, 2018). So, students feel it is enjoyable and less stressful to attain this element of vocabulary knowledge after listening to music, and their positive learning gains can be retained several weeks later. Second, music adds to the understanding of lyrics by allowing listeners to sense positive and negative signals while listening. According to Thompson and Russo (2004) research, the sound of music influences the understanding of meaning and emotion in song lyrics, and it can influence the perceived meaningfulness of lyrics with repeated song exposure. As a result, vocabulary knowledge of meaning connections also results in effective improvement for all students after listening to songs, and this will probably be remembered 4 weeks later. However, there was little statistically noteworthy difference between the pretest, intermediate, and delayed posttest results across all students’ knowledge of grammar recognition. Students were not consciously aware of the function and characteristics of grammar (Hulstijn, 2003), so there was no progress in grammar recognition for all students after listening to songs.

### Limitations and Future Directions

Several restrictions should be considered while evaluating our data. First, only two pop songs were employed in this study; thus, more research with more songs and various types of songs is encouraged to analyze which types of songs best accelerate vocabulary acquisition.

Second, in the current research, all the participants were college students with most having a restricted vocabulary of 4,000–5,900 words. Besides, an earlier study found a favorable correlation between vocabulary size and learning gains in accidental vocabulary acquisition Vidal (2003, 2011). Thus, to acquire a better understanding of how listening to songs could benefit different levels of L2 learners, future research should investigate incidental vocabulary learning while listening to songs, particularly with individuals with smaller or larger vocabulary sizes.

Third, vocabulary knowledge covers different areas, such as a word’s spoken or written form, collocations, semantic network of associations, etc. (Nation, 1990). However, this study focuses solely on word recognition, meaning recall, and grammar. As a result, it would be beneficial to study other aspects of vocabulary knowledge after listening to songs in future studies.

Fourth, learning efficiency is a metric for academic accomplishment that takes into accounts both performance and the amount of time a student spends learning. However, the difference in learning efficiency among students with different degrees of prior vocabulary knowledge was not considered in this research (Xiongfei et al., 2021; Khan, 2022; Mehmood et al., 2022). It is worthwhile to investigate in future language learning studies.

Fifth, in the present research, only the learner-internal factor was considered during the investigation. However, there are many other factors of individual difference to examine, such as working memory, motivation, age, enjoyment, etc. (see Elgort and Warren, 2014; Lee and Pulido, 2017; Koda and Miller, 2018; Malone, 2018; Xiongfei et al., 2019; Bahadur et al., 2020; Pitafi et al., 2020). Some studies have looked at the role of working memory in reading and found a strong link to reading comprehension (Varol and Ercetin, 2016). Thus, it would be useful to investigate these different factors of individual differences in future incidental vocabulary research, especially in listening.

## Conclusion

The current research provides extensive empirical support for the possibility of incidental vocabulary learning through listening to songs. The results demonstrate that learners may acquire the spoken and meaning forms, and perform grammatical identification of single-word items by listening to songs. The effect of word recognition, on the other hand, was superior to the other two elements, and it was maintained 4 weeks later. Furthermore, repeated listening to English songs may promote vocabulary learning, but it was surprising to find that the more times a song was heard, this did not guarantee a greater total amount of vocabulary information being obtained. As a result, the data show that listening to songs three times (with exposure frequency ranging from three to nine times) was the most beneficial for L2 learners, with word recognition information likely to be preserved after 4 weeks. When the learner-internal component was taken into account, it was surprisingly found that low, intermediate, and high-proficiency learners all showed a beneficial influence on vocabulary knowledge of work-form recognition and meaning connection after listening to songs. As music has the ability to spark learners’ interest and make learning happen subconsciously, listening to songs can be an excellent way to learn new vocabulary, particularly for low-proficiency students who are usually reluctant to attend English learning activities.

## Pedagogical Implications

This new research provides further empirical evidence supporting the favorable impact of listening to songs on vocabulary learning. It is advisable, then, for language teachers to use “listening to English songs” as another teaching strategy to improve students’ vocabulary knowledge in the classroom. This could also have the double impact of exposing students to the style of information that best matches their learning preferences, and encourage them to learn outside the classroom using their chosen source of input, which is especially important for low-level students who are not interested in traditional vocabulary learning and struggle to learn receptive knowledge of word recognition and meaning recall. Furthermore, the repeated concurrence of single-words from three to nine times throughout the song may benefit learners most. After repeated exposure, the three dimensions of vocabulary knowledge (word-recognition, meaning recall, and grammar) may improve after listening, and word recognition will remain 4 weeks later. As a result, instructors and learners can use “listening to songs three times” as a criterion for efficient vocabulary teaching and learning.

## Data Availability Statement

The raw data supporting the conclusions of this article will be made available by the authors, without undue reservation.

## Ethics Statement

Ethical approval was not provided for this study on human participants because it was not mandatory to take ethical approval first, however, all procedures were followed by the ethical guidelines of the university and international institutions. Written informed consent to participate in this study was provided by the participants’ legal guardian/next of kin.

## Author Contributions

KN constructed the manuscript overall. JF was responsible for conducting experiments. HR helped in the write-up. GZ helped in improving, data analysis, and proofreading. All authors contributed to the article and approved the submitted version.

## Conflict of Interest

The authors declare that the research was conducted in the absence of any commercial or financial relationships that could be construed as a potential conflict of interest.

## Publisher’s Note

All claims expressed in this article are solely those of the authors and do not necessarily represent those of their affiliated organizations, or those of the publisher, the editors and the reviewers. Any product that may be evaluated in this article, or claim that may be made by its manufacturer, is not guaranteed or endorsed by the publisher.

## Footnotes

1
http://www.victoria.ac.nz/lals/staff/paul-nation.aspx
